# Transcriptional loops meet chromatin: a dual-layer network controls white–opaque switching in *Candida albicans*

**DOI:** 10.1111/j.1365-2958.2009.06772.x

**Published:** 2009-07-06

**Authors:** Denes Hnisz, Tobias Schwarzmüller, Karl Kuchler

**Affiliations:** Medical University Vienna, Christian Doppler Laboratory for Infection Biology, Max F. Perutz Laboratories, Campus Vienna BiocenterA-1030 Vienna, Austria

## Abstract

The human pathogen *Candida albicans* is able to undergo a reversible switch between two distinct cell types called white and opaque, which are considered different transcriptional states of cells harbouring identical genomes. The present model of switching regulation includes the bistable expression of a master switch gene that is controlled by multiple transcriptional feedback loops. Here, we show that chromatin-modifying enzymes constitute an additional important regulatory layer of morphogenetic switching. We identify eight chromatin modifiers as switching modulators. Extensive epistasis analysis maps them into at least two independent signalling pathways overlaying the known transcriptional network. Interestingly, we identify the conserved Set3/Hos2 histone deacetylase complex as a key regulator relying on the methylation status of histone H3 lysine 4 for switching modulation. Furthermore, we demonstrate that opaque to white switching is facilitated by the presence of adenine *in vitro*, but adenine has no effect on switching once the Set3/Hos2 complex is disrupted. Our observations postulate that chromatin modifications may serve as a means to integrate environmental or host stimuli through the underlying transcriptional circuits to determine cell fate in *C. albicans*.

## Introduction

Individual cells in a genetically homogenous microbial culture may display different phenotypic characteristics. Such cell-to-cell variability is suggested to enhance the ability of microbial populations to adapt to a wide range of environmental stimuli, which, in the case of pathogens, may represent a strategy to evade host defences (Avery, 2006). The fungal pathogen *Candida albicans* displays a remarkable spectrum of heritable morphogenetic variations which is considered a major factor in the transition from a harmless commensal to a systemic pathogen of its human host (Whiteway and Bachewich, 2007). An intriguing and unique ability of *C. albicans* is to form two distinct cell types: the so-called white and opaque phases. White and opaque cells contain the same genome, yet they differ in cellular morphology, colony shape, gene expression profile and virulence properties. In addition, white cells are unable to mate, whereas opaque cells are mating-competent (Bennett and Johnson, 2005).

White–opaque switching is an epigenetic phenomenon that was already described some 20 years ago (Slutsky *et al*., 1987), but the underlying molecular mechanisms have been only recently investigated. The white and opaque phases are heritable for many generations and switching between both phases is reversible, occurring at a frequency of one per ∼10^3^−10^4^ cell divisions (Rikkerink *et al*., 1988). The regulation of switching is believed to be transcriptional, and several transcription factors involved have been identified. *C. albicans* is obligatory diploid and harbours a mating type-like locus (*MTL*) holding two alleles, ‘**a**’ and ‘α’. Hence, the possible *MTL* configurations include *MTL***a**/**a,***MTL*α/α and *MTL***a**/α (Hull and Johnson, 1999). A heterodimeric **a**/α repressor encoded by the **a** and α alleles respectively, locks *MTL* heterozygous cells in the white phase (Miller and Johnson, 2002) by repressing *WOR1*, the master opaque-promoting factor (Zordan *et al*., 2006). *MTL* homozygous cells lack the **a**/α repressor, and are thus permissive to switching. In *MTL***a**/**a** or *MTL*α/α white cells, *WOR1* is expressed at a very low level, and high-level expression of *WOR1* is required for the conversion to the opaque phase (Huang *et al*., 2006; Srikantha *et al*., 2006; Zordan *et al*., 2006). By contrast, the transcription factor Efg1 is enriched in white cells and is required for maintenance of the white phase (Sonneborn *et al*., 1999; Srikantha *et al*., 2000). According to the current model, stochastic increase in Wor1 levels drive the transition from the white to the opaque phase. Furthermore, Wor1 autoregulates its own expression, facilitates expression of its cofactor *WOR2*, and represses *EFG1* both directly and indirectly through promoting the expression of *CZF1*, a repressor of *EFG1*. As *EFG1* is a putative repressor of *WOR2*, *WOR1* thus co-ordinates three positive feedback loops to ensure high Wor1 levels, explaining the heritability of the opaque phase (Zordan *et al*., 2007). In addition, the histone deacetylases Hda1 and Rpd3 have been implicated in the regulation of white–opaque switching (Klar *et al*., 2001; Srikantha *et al*., 2001) but their precise role remains to be clarified.

In this work, we show that a complex dual-layer network, comprising of transcriptional regulators and chromatin-modifying enzymes, determines cellular identity in *C. albicans*. Our results experimentally confirm previous suggestions that cellular shape and phase-specific genes are regulated at different branching points of the transcriptional circuit, and that the genetic information affecting phase commitment converges at the *WOR1* locus. Importantly, we identify eight genes encoding putative histone-modifying enzymes as novel modulators of white–opaque switching in *C. albicans*. An extensive epistasis analysis maps various histone-modifiers into the transcriptional circuit. Strikingly, we show that the Set3/Hos2 histone deacetylase complex is a key regulator of *WOR1* expression, and thus conversion to the opaque phase. Furthermore, we provide genetic evidence that the newly identified Set3/Hos2 defines a pathway depending on histone H3 lysine 4 (H3K4) methylation for switching regulation. Finally, we identify adenine as novel environmental factor facilitating opaque to white conversion, and demonstrate that the regulatory effect of adenine on switching requires *SET3*. We propose a comprehensive model whereby chromatin modifiers constitute a layer of regulation modulating the transcriptional circuits to trigger switching. Chromatin modification offers a possible mechanism to integrate environmental stimuli, contrary to the current models that explain morphogenetic switching as a purely stochastic process. Moreover, we postulate that the dependence of the Set3/Hos2 complex on H3K4 methylation at certain loci may be an evolutionary conserved mechanism among other eukaryotic taxa.

## Results

### *WOR1* acts downstream of *EFG1* in phase commitment, while *EFG1* acts downstream of *WOR1* in morphology determination

The white and opaque cell types of *C. albicans* are distinguished based on four criteria. (i) Cellular morphology: white cells have a round shape; opaque cells are larger and elongated (Slutsky *et al*., 1987). (ii) Colony appearance: white cells form white, dome-shaped colonies on solid agar, while opaque cells form larger, flattened colonies that are stained pink on media containing Phloxin B (Slutsky *et al*., 1987). (iii) Gene expression profile: about 400 genes are regulated differentially in the two phases (Lan *et al*., 2002). For diagnostic purposes, the white-specific genes *WH11* (Srikantha and Soll, 1993) and *EFG1* (Sonneborn *et al*., 1999), as well as opaque-specific genes *OP4* (Morrow *et al*., 1993) and *SAP1* (Morrow *et al*., 1992) are commonly used. (iv) Mating competence: white cells are mating incompetent, whereas opaque cells can mate with opaque cells of the opposite mating type (Miller and Johnson, 2002).

Previous studies established *WOR1* as the master regulator of the opaque phase. Deletion of *WOR1* locks *MTL***a**/**a** or *MTL*α/α cells in the white phase, whereas ectopic overexpression of *WOR1* results in the conversion to the opaque phase (Huang *et al*., 2006; Zordan *et al*., 2006). On the other hand, *MTL* homozygous *efg1*Δ/Δ cells predominantly exist in the opaque phase, while ectopic *EFG1* expression drives opaque to white conversion (Sonneborn *et al*., 1999). Recently, *EFG1* was suggested to promote the white phase by repressing *WOR2*, a cofactor of *WOR1* (Zordan *et al*., 2007). In addition, *EFG1* was proposed to act downstream of the switching event to regulate cellular morphology (Srikantha *et al*., 2000).

**Fig. 1:**
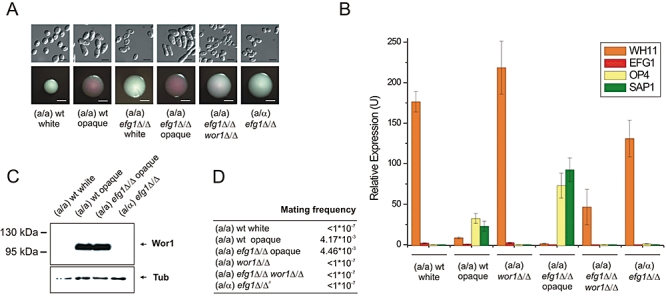
*WOR1* acts downstream of *EFG1* in phase commitment, while *EFG1* acts downstream of *WOR1* in morphology determination. A. Colony and cellular morphologies on modified Lee's medium containing 5 μg ml^−1^ Phloxin B. Scale bars correspond to 5 μm (upper panel) and 2 mm (lower panel). B. qRT-PCR analysis of phase-specific mRNA transcripts. *WH11*, *EFG1* (white-specific) and *OP4*, *SAP1* (opaque-specific) transcript levels were normalized to the transcript level of *PAT1* (Zordan *et al*., 2006). qRT-PCR reactions were performed in triplicates and cDNA isolated from two independent cultures were analysed. Data are shown as mean ± SD. C. Immunoblot analysis confirms that *WOR1* is repressed by *EFG1* and the **a**/α repressor. Tubulin indicates equivalent loading. D. *WOR1* is required for mating. Quantitative mating assays were performed with an opaque phase *MTL*α/α tester strain. At least two independent experiments per genotype were performed yielding qualitatively similar results. Values are shown of one representative experiment. #: tested with both an *MTL***a**/**a** and an *MTL*α/α tester strain.

In order to experimentally verify the latter two suggestions, we created an *efg1*Δ/Δ*wor1*Δ/Δ double mutant in an *MTL***a**/**a** background. The *MTL***a**/**a***efg1*Δ/Δ*wor1*Δ/Δ mutant displayed an elongated cell shape, albeit shorter than wild type opaque cells, similar to the rare *MTL***a**/**a***efg1*Δ/Δ white cells as well as *MTL***a**/α*efg1*Δ/Δ cells. In addition, the *MTL***a**/**a***efg1*Δ/Δ*wor1*Δ/Δ mutant formed large, flattened colonies appearing light pink on Phloxin B agar, intermediate to the white and pink colour of wild type white and opaque cells respectively. Conversely, *MTL***a**/**a***efg1*Δ/Δ white isolates and *MTL***a**/α*efg1*Δ/Δ cells were white on Phloxin B plates (Fig. 1A). We inspected over 2000 colonies and all of them displayed the described morphology.

Next, we found that the *efg1*Δ/Δ*wor1*Δ/Δ double mutant expressed the white-specific transcript *WH11* similar to wild type white and *wor1*Δ/Δ cells (the latter being locked in the white phase). Conversely, the opaque-specific transcripts *OP4* and *SAP1* were virtually undetectable. *MTL***a**/α*efg1*Δ/Δ cells also showed a white-phase expression profile (Fig. 1B). These data are in accordance with previous publications and suggest that in switching-permissive cells, loss of *EFG1* results in the formation of true opaque cells due to the upregulation of *WOR1*, which we directly confirmed by immunoblotting (Fig. 1C). As expected, the **a**/α repressor still inhibits *WOR1* expression in *MTL***a**/α*efg1*Δ/Δ cells, thus locking cells in a white-like phase (Fig. 1A–C). Therefore, *EFG1* indeed promotes the white phase by directly or indirectly repressing *WOR1*.

We also tested the mating ability of the *MTL***a**/**a***efg1*Δ/Δ*wor1*Δ/Δ double mutant, and found that its mating competence was as negligible as that of wild type white cells (Fig. 1D). Therefore, *MTL***a**/**a***efg1*Δ/Δ*wor1*Δ/Δ cells are functionally white and express white-specific genes. Nevertheless, they show an elongated morphology distinguishable from wild type white cells. These data demonstrate that *WOR1* acts downstream of *EFG1* in phase commitment, while *EFG1* acts downstream of *WOR1* in morphology determination.

### Several histone-modifying genes modulate white–opaque switching

Although transcription factors are known to regulate white–opaque conversion, neither a rearrangement in DNA sequences nor any modification of chromatin has been associated with switching. Notably, lack of the histone deacetylase genes *HDA1* and *RPD3* modify frequencies of switching (Klar *et al*., 2001; Srikantha *et al*., 2001). Therefore, we decided to analyse the contribution of histone-modifying enzymes to phase transitions in a comprehensive way. We analysed the genome of the related fungal species *Saccharomyces cerevisiae* (http://www.yeastgenome.org) to identify open reading frames (ORFs) encoding putative histone modifiers (acetyltransferases, deacetylases, methyltransferases and dephosphorylases) either as regulatory or catalytic subunits of larger protein complexes. Out of some 90 genes, we selected only those encoding catalytic subunits, yielding a total of 23 genes (including one additional ORF: *SET3*). blast searches (http://www.ncbi.nlm.nih.gov/blast) against the *C. albicans* genome identified all potential orthologues, revealing that *S. cerevisiae* histone modifiers are highly conserved in *C. albicans*. Subsequently, we constructed homozygous deletion mutants of the listed ORFs in an *MTL***a**/**a***C. albicans* strain. Out of 23 candidates, we successfully created homozygous deletion strains of 18 genes. The identified ORFs, their predicted functions, the blast*E*-values and whether a deletion mutant was created are listed in Table S1.

Next, we analysed the effect of gene deletions on the frequency of white to opaque conversion using quantitative switching assays. Briefly, pure white cultures were plated on Phloxin B plates, and the frequency of opaque colonies or colonies containing at least one opaque sector was scored (as monitored by colony morphology and microscopy). Knock-out mutants showing significant alterations compared to the background strain are listed in Table 1. *C. albicans* genes whose deletion facilitated the formation of opaque colonies or sectors included *SET1*, a H3K4 methyltransferase (Roguev *et al*., 2001; Raman *et al*., 2006) required for gene silencing at telomeres and rDNA sequences in *S. cerevisiae* (Nislow *et al*., 1997); *HDA1*, a histone deacetylase (Carmen *et al*., 1996) acting as a global repressor of transcription in *S. cerevisiae* (Rundlett *et al*., 1996); *HDA1* served as a control in our gene set, because its loss was previously shown to increase the frequency of opaque formation (Klar *et al*., 2001); and *RPD31*, one of the two orthologues of yeast *RPD3*, a histone deacetylase involved in transcriptional repression in *S. cerevisiae* (Rundlett *et al*. (1996). The genome of *C. albicans* harbours two potential orthologues of *RPD3* designated *RPD3* and *RPD31* (Table S1). Interestingly, a deletion of *RPD3* has similar effects on the white to opaque switching frequency (Srikantha *et al*., 2001). Genes whose deletion significantly decreased opaque conversion relative to wild type included: *SET3*, an essential component of the Set3 histone deacetylase complex involved in the suppression of meiotic genes in *S. cerevisiae* (Pijnappel *et al*., 2001); *HOS2*, a histone deacetylase and subunit of the Set3 complex (Pijnappel *et al*., 2001) required for gene activity in *S. cerevisiae* (Wang *et al*., 2002); *HST2*, a histone deacetylase similar to *SIR2* (Landry *et al*., 2000) required for centromeric and rDNA silencing in *S. cerevisiae* (Durand-Dubief *et al*., 2007); and *NAT4*, an acetyltransferase mediating histone H4 and H2A acetylation (Song *et al*., 2003).

**Table 1:** Histone-modifier genes modulate white–opaque switching.

	White → opaque		Opaque → white	
Strain	Switch (%)	*n*	Switch (%)	*n*
wt (**a**/α)	0 ± 0	1808	–	–
wt (**a**/**a**)	11.3 ± 1.9	1113	10.2 ± 1.1	1089
*set1*Δ/Δ	19.5 ± 4.5*	863	10.0 ± 4.5	1886
*hda1*Δ/Δ	30.8 ± 13.2*	2328	10.8 ± 5.4	1320
*rpd31*Δ/Δ	32.2 ± 0.7**	1289	12.5 ± 1.4	800
*set3*Δ/Δ	1.8 ± 0.3**	1352	27.6 ± 2.4**a	743
*hos2*Δ/Δ	1.5 ± 0.2**	1539	23.4 ± 2.2*a	1495
*hst2*Δ/Δ	0.4 ± 0.4**	1994	19.0 ± 14.0	1272
*nat4*Δ/Δ	1.4 ± 0.6**	2006	34.9 ± 13.7*	1171
*hst1*Δ/Δ	14.6 ± 4.8	2038	3.8 ± 0.4**	1270
*pho13*Δ/Δ	10.5 ± 1.1	807	51.1 ± 7.9**	1033

a. Informative value (see text).

* *P* < 0.05 and

** *P* < 0.005 relative to wild type (Student's *t*-test).

Quantitative white to opaque (left panel) and opaque to white (right panel) switching assays were performed with multiple homozygous deletion mutants. The percentages represent the fraction of colonies that showed an alteration of the original phenotype. The gene deletions were constructed in the wild type *MTL***a**/**a** background strain (second row). As expected, wild type *MTL***a**/α strains are locked in the white phase. Data are displayed as a mean ± SD as well as the total number of colonies scored in three independent experiments carried out with the same strain.

Furthermore, we analysed the impact of the deletions on the heritability of the opaque phase using quantitative switching assays. In these assays, opaque phase cultures were plated on Phloxin B agar, and the arising frequency of pure white colonies and colonies containing at least one white sector was scored (Table 1). Genes whose deletion increased the heritability of the opaque phase (i.e. displaying a lower frequency of conversion to white than wild type) included *HST1*, a histone deacetylase, a nonessential subunit of the Set3 complex (Pijnappel *et al*., 2001), as well as an essential subunit of the Sum1/Rfm1/Hst1 complex, which functions as a repressor of sporulation-specific genes in *S. cerevisiae* (Xie *et al*., 1999). Genes whose deletion destabilized the opaque phase (i.e. the deletion mutant showed a higher frequency of conversion to white than wild type) included *PHO13*, a phosphatase dephosporylating H2A *in vitro* (Tuleva *et al*., 1998) and implicated in carbohydrate metabolism in *S. cerevisiae* (Van Vleet *et al*., 2007) and *NAT4* (see above).

Surprisingly, the loss of either *SET3* or *HOS2* led to an unexpected phenotype: the opaque colonies of the *set3*Δ/Δ and *hos2*Δ/Δ mutants displayed filamentous growth (D. Hnisz and K. Kuchler, in preparation). We assayed the opaque to white switching frequencies of these filamenting mutants, and found an increase in the conversion to the white phase when compared with wild type cultures (Table 1); however, these data should be interpreted with caution, because the filaments could not be reliably fragmented into individual colony forming units with our method (see *Experimental procedures*). Moreover, a lack of *SET3* or *HOS2* is likely to exert pleiotropic effects on both white–opaque switching and filamentation, i.e. by affecting two distinct transcriptional programmes, whose putative cross-talk would inherently impact the scoring method.

**Fig. 2:**
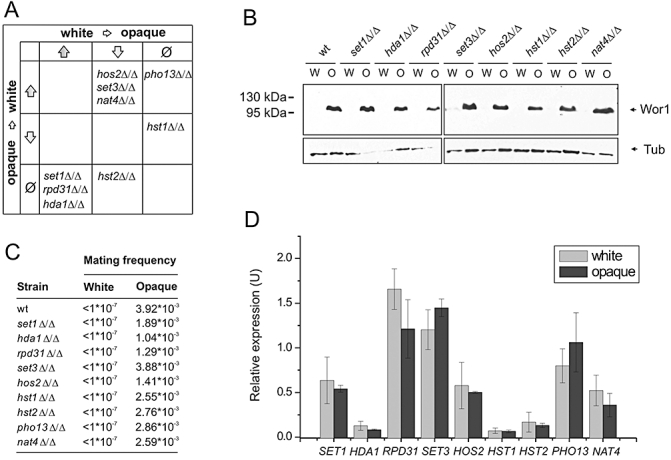
Histone modifiers act upstream of *WOR1*. A. Functional categories of single gene deletions on white–opaque switching. B. Immunoblot analysis demonstrates that Wor1 is expressed in a similar pattern in wild type and mutant white (W) and opaque (O) cultures. Tubulin indicates equivalent loading. C. Mating competence is differentially regulated in single mutant cells similar to wild type. Quantitative mating assays were performed with an opaque phase *MTL*α/α tester strain. At least two independent experiments per genotype were performed giving qualitatively similar results. Values are shown of one representative experiment. D. Transcript levels of histone modifiers are phase-independent. qRT-PCR was performed in triplicates and cDNA isolated from two independent cultures were analysed. Transcript levels are normalized to *PAT1*. Data are shown as mean ± SD.

To confirm the switching data, we created a second independent set of deletion mutants of all genes showing an effect on switching and repeated the quantitative switching assays in both directions. In all cases, the independent deletion strains qualitatively reproduced all relevant phenotypes of the first deletion strains (data not shown). Furthermore, we complemented the deletions of *SET3*, *HOS2*, *NAT4* and *HST2* which were the key genes of our further analyses. The complemented strains displayed switching frequencies comparable to wild type (Table S8). As summarized in Fig. 2A, these results show that histone-modifying enzymes of various classes can modulate white–opaque switching in multiple ways. However, where and how histone modifiers modulate phase conversion is not yet clear.

### Histone modifiers act upstream of *WOR1*

Previous work established that the formation of mating-competent opaque cells requires Wor1 (see above). Indeed, we were unable to detect Wor1 by immunoblotting in any of the investigated white phase single mutant cultures. By contrast, opaque phase mutant cells expressed Wor1 at levels comparable to those present in wild type opaque strains (Fig. 2B). Furthermore, white and opaque phase single deletion mutants exhibited mating competence comparable to wild type white and opaque strains respectively (Fig. 2C). These results suggest that histone modifiers act either upstream or at the level of *WOR1* expression.

To address whether phase-specific expression of genes is responsible for phase changes, we performed quantitative RT-PCR to compare expression levels of switching modulators in the white and opaque phases (Fig. 2D). We failed to detect any significant differences of transcript levels between the two phases, neither in our *MTL***a**/**a** background strain (Fig. 2D) nor in the independent *MTL***a**/**a** clinical isolate L26 (data not shown). These results demonstrate that the activities rather then expression levels of histone modifiers modulate the outcome of the transcriptional regulatory circuit(s), which converge(s) at the master switch locus *WOR1*.

### Epistasis of *SET3*, *HOS2*, *HST2*, *NAT4* and *EFG1* reveals multiple pathways

Interestingly, although the genes have different molecular functions, deletion of *SET3*, *HOS2*, *HST2* and *NAT4* all reduced the switching frequency about 5–10-fold from white to opaque (see above). Altered chromatin state can influence DNA accessibility to non-histone proteins such as the transcriptional machinery or transcription factors can recruit chromatin-modifying enzymes to facilitate their activities (Kouzarides, 2007). For instance, the *C. albicans* transcription factor Efg1 is a basic helix–loop–helix protein displaying DNA-binding activity *in vitro* has also been implicated in phase switching (Stoldt *et al*., 1997). To elucidate whether *SET3*, *HOS2*, *HST2* or *NAT4* modulate white to opaque switching in concert with *EFG1*, we constructed *efg1*Δ/Δ*hos2*Δ/Δ, *efg1*Δ/Δ*set3*Δ/Δ, *efg1*Δ/Δ*hst2*Δ/Δ and *efg1*Δ/Δ*nat4*Δ/Δ double mutants, and performed epistasis analysis by comparing their switching frequencies with those of the corresponding single mutants (Tables 1 and 2). The phases were verified by colony morphology, microscopy (data not shown) and quantitative mating assays (Table S5). The results were as follows: *efg1*Δ/Δ: 97.7%, *set3*Δ/Δ: 1.8%, *hos2*Δ/Δ: 1.5%, *hst2*Δ/Δ: 0.4% and *nat4*Δ/Δ: 1.4%, whereas for the double mutants: *efg1*Δ/Δ*set3*Δ/Δ: 3.0%, *efg1*Δ/Δ*hos2*Δ/Δ: 3.6%, *efg1*Δ/Δ*hst2*Δ/Δ: 87.9% and *efg1*Δ/Δ*nat4*Δ/Δ: 89.2% (Tables 1 and 2). Hence, loss of *EFG1* is epistatic to the deletion of either *HST2* or *NAT4*, whereas *HOS2* or *SET3* deletion qualitatively suppresses the loss of *EFG1*. In other words, although the repression of *EFG1* on *WOR1* is relieved, stable high-level expression of *WOR1* still requires both *SET3* and *HOS2*. On the other hand, *HST2* and *NAT4* are likely to exert their effect in a transcriptional loop converging at the *WOR1* locus either at the level of *EFG1* or upstream of it. Notably, the opposite switching frequencies from opaque to white showed a similar epistasis (right panel, Table 2). As a further control, we restored the *SET3* and *HOS2* ORFs in *efg1*Δ/Δ*set3*Δ/Δ and *efg1*Δ/Δ*hos2*Δ/Δ cells, respectively, and found that the complemented mutants showed switching frequencies comparable to the *efg1*Δ/Δ mutant (Table S8).

**Table 2:** Epistasis analysis of *SET3*, *HOS2*, *NAT4*, *HST2* and *EFG1*.

	White → opaque		Opaque → white	
Strain	Switch (%)	*n*	Switch (%)	*n*
wt	11.3 ± 1.9	1113	10.2 ± 1.1	1089
*efg1*Δ/Δ	97.7 ± 1.0	1110	0.6 ± 0.8	1576
*efg1*Δ/Δ*hst2*Δ/Δ	87.9 ± 20.1	940	1.9 ± 2.6	1969
*efg1*Δ/Δ*nat4*Δ/Δ	89.2 ± 10.7	812	0.3 ± 0.6	1568
*efg1*Δ/Δ*set3*Δ/Δ	3.0 ± 1.6	2685	12.9 ± 6.7	1299
*efg1*Δ/Δ*hos2*Δ/Δ	3.6 ± 1.3	1148	5.2 ± 6.3	1061
*efg1*Δ/Δ*hst2*Δ/Δ*hos2*Δ/Δ	5.6 ± 3.6	1117	5.5 ± 3.7	599
*efg1*Δ/Δ*nat4*Δ/Δ*set3*Δ/Δ	2.7 ± 1.3	1322	10.4 ± 6.9	906

Quantitative white to opaque (left panel) and opaque to white (right panel) switching assays were performed with multiple homozygous deletion mutants. The percentages represent the fraction of colonies that showed an alteration of the original phenotype. All strains are *MTL***a**/**a** strains. Data are displayed as a mean ± SD as well as the total number of colonies scored in three independent experiments carried out with the same strain.

To verify that *HOS2* and *SET3* indeed act in an independent pathway of either *NAT4* or *HST2,* we tested the switching frequencies of *efg1*Δ/Δ*hst2*Δ/Δ*hos2*Δ/Δ and *efg1*Δ/Δ*nat4*Δ/Δ*set3*Δ/Δ triple mutants in both switching directions. As predicted, deletion of *NAT4* in an *efg1*Δ/Δ*set3*Δ/Δ mutant, and deletion of *HST2* in an *efg1*Δ/Δ*hos2*Δ/Δ mutant had no significant effect on switching frequencies when compared with the respective double deletion strains (Table 2). In summary, the epistasis analysis revealed at least two independent regulatory pathways affecting the transcriptional loops controlling morphogenetic switching.

### Loss of *SET3* or *HOS2* suppresses deletion of *RPD31* or *HDA1*

The *S. cerevisiae* orthologues of Hos2, Hda1 and Rpd31 are histone deacetylases catalytically active on multiple acetylated lysine residues of core histones (Pijnappel *et al*., 2001; Suka *et al*., 2001; Wu *et al*., 2001). Set3 is an integral subunit of the Set3/Hos2 deacetylase complex (Pijnappel *et al*., 2001). To address whether there is a division of labour between deacetylase complexes in the regulation of *C. albicans* white–opaque switching, we created a series of double deletion strains and compared their switching frequencies with those of single deletion mutants. As shown in Tables 1 and 3, deletion of *SET3* is epistatic to the loss of *HDA1* and *RPD31,* and deletion of *HOS2* is epistatic to the deletion of *RPD31*. Phases were verified by colony morphology, microscopy (data not shown) and quantitative mating assays (Table S6). These results support the notion that Set3 and Hos2 act in a complex in *C. albicans* functioning as a downstream regulator of white–opaque switching.

### Loss of H3K4 methylation suppresses the effect of the deletion of *SET3* or *HOS2*

**Fig. 3:**
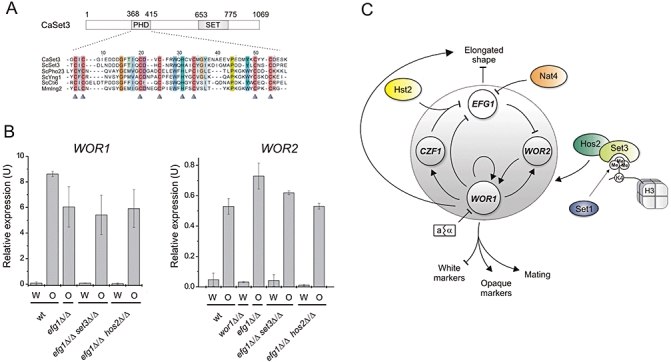
The Set3/Hos2 complex is a key regulator of white–opaque switching. A. The PHD finger of CaSET3. The amino acid sequence was aligned to the PHD fingers of ScSet3, ScPho23, ScYng1, ScCti6 and MmIng2 that were shown to bind H3K4me3 specifically *in vitro* (Shi *et al*., 2006; 2007). Colours indicate homologous residues. Arrowheads highlight the residues of the characteristic Cys_4_-His-Cys_3_ Zn^2+^ co-ordination motif. B. qRT-PCR analysis of *WOR1* and *WOR2* expression in Set3/Hos2-pathway mutants. Deletion of either *SET3* or *HOS2* in an *efg1*Δ/Δ background does not cause significant changes in the steady-state transcription level of either *WOR1* or *WOR2*. The mRNA levels are normalized to *PAT1*. qRT-PCR reactions were performed in triplicates and cDNA isolated from two independent cultures were analysed. Data are shown as mean ± SD. C. Dual-layer model of the regulation of white–opaque switching in *C. albicans*. The dotted grey circle denotes the transcriptional circuit as described (Zordan *et al*., 2007). White and opaque enriched regulators are shown in white and grey respectively. Coloured elements represent histone-modifying pathways modulating the output of the transcriptional circuit.

Where and how is the Set3/Hos2 complex recruited? Inspection of the CaSet3 primary sequence revealed two characteristic domains: a SET and a PHD (Plant HomeoDomain) domain (Fig. 3A). This domain architecture is conserved among many genes implicated in epigenetic regulation, including *ASH1* and *Thrithorax* in *Drosophila* (Stassen *et al*., 1995; Tripoulas *et al*., 1996). The SET domains have two functions: methyltransferase activity acting on histones (Rea *et al*., 2000) or other non-histone substrates, and they may serve as protein–protein interaction surfaces (Rozenblatt-Rosen *et al*., 1998). The PHD finger is a specialized methyl-lysine binding domain found in various proteins ‘reading’ histone marks (Shi *et al*., 2006). Recently, the purified PHD finger of ScSet3 was shown to preferentially bind trimethylated H3K4 (Shi *et al*., 2007). Notably, *CaSET1* appears as the only *C. albicans* methyltransferase modifying H3K4, and its deletion results in a complete loss of H3K4 methylation (Raman *et al*., 2006).

To address whether the Set3/Hos2 complex requires H3K4 methylation for the regulation of white–opaque switching in *C. albicans*, we compared the switching frequencies of *set1*Δ/Δ*set3*Δ/Δ and *set1*Δ/Δ*hos2*Δ/Δ double mutants to those of the respective single deletion strains (Tables 1 and 4). Strikingly, the absence of *SET1* almost completely suppressed the loss of *SET3* or *HOS2* (white to opaque switching frequencies: *set1*Δ/Δ: 19.5%, *hos2*Δ/Δ: 1.5%, *set3*Δ/Δ: 1.8%, *set1*Δ/Δ*set3*Δ/Δ 10.5%, *set1*Δ/Δ*hos2*Δ/Δ 12.8%). To verify the rescue effect, we performed an epistasis analysis of *SET1* and *HOS2* in a genetic background where the transcriptional feedback from *EFG1* towards *WOR1* is disrupted. Therefore, we compared the white to opaque switching frequencies of *efg1*Δ/Δ, *efg1*Δ/Δ*set1*Δ/Δ, *efg1*Δ/Δ*hos2*Δ/Δ and *efg1*Δ/Δ*set1*Δ/Δ*hos2*Δ/Δ mutants (Tables 1, 2 and 4). White *efg1*Δ/Δ cells converted at high frequencies to opaque (97.7%, Table 2). *efg1*Δ/Δ*set1*Δ/Δ cells almost exclusively existed in the opaque phase, as we failed to isolate single white phase colonies of this mutant. *efg1*Δ/Δ*hos2*Δ/Δ white cells converted at low frequencies to opaque cells (3.6%, Table 2). As expected, *efg1*Δ/Δ*set1*Δ/Δ*hos2*Δ/Δ white cells also readily convert to the opaque phase (80.6%, Table 4), while the opaque phase appears as stable as in *efg1*Δ/Δ and *efg1*Δ/Δ*set1*Δ/Δ opaque isolates (Tables 2 and 4). The phases were verified by colony morphology, microscopy (data not shown) and quantitative mating assays (Table S7).

**Table 4:** Loss of H3K4 methylation suppresses the effects of *SET3* or *HOS2* deletions.

	White → opaque		Opaque → white	
Strain	Switch (%)	*n*	Switch (%)	*n*
wt	11.6 ± 4.5	560	21.8 ± 9.5	609
*set1*Δ/Δ*hos2*Δ/Δ	12.8 ± 2.7	1303	NA	–
*set1*Δ/Δ*set3*Δ/Δ	10.5 ± 4.4	1108	NA	–
*efg1*Δ/Δ*set1*Δ/Δ	NA	–	0.2 ± 0.3	1636
*efg1*Δ/Δ*set1*Δ/Δ*hos2*Δ/Δ	80.6 ± 7.5	1318	1.4 ± 1.5	1730
*wor1*Δ/Δ	0 ± 0	1661	NA	–
*wor1*Δ/Δ*set1*Δ/Δ	0 ± 0	1458	NA	–
*wor1*Δ/Δ*set3*Δ/Δ	0 ± 0	1734	NA	–
*wor1*Δ/Δ*hos2*Δ/Δ	0 ± 0	1079	NA	–
*wor1*Δ/Δ*set1*Δ/Δ*hos2*Δ/Δ	0 ± 0	1662	NA	–

Quantitative white to opaque (left panel) and opaque to white (right panel) switching assays were performed with multiple homozygous deletion mutants. The percentages represent the fraction of colonies that showed an alteration of the original phenotype. All strains are *MTL***a**/**a** strains. Data are displayed as a mean ± SD as well as the total number of colonies scored in three independent experiments carried out with the same strain.

NA, not assayed.

As deletion of either *SET3* or *HOS2* was epistatic to the deletion of *EFG1* in switching modulation (Table 3), we sought evidence that the newly identified Set3/Hos2 pathway indeed regulates *WOR1* expression to drive phenotypic switching. As shown in Table 4, deletion of *WOR1* in *set1*Δ/Δ, *set3*Δ/Δ, *hos2*Δ/Δ and *set1*Δ/Δ*hos2*Δ/Δ mutants locks cells in the white phase, supporting that the Set3/Hos2 pathway mediates regulatory input upstream or at the level of *WOR1* expression. To test whether the disruption of the pathway is reflected in the steady state transcript levels of *WOR1*, we performed quantitative real-time PCR analysis, but found no difference in *WOR1* mRNA levels between wild type, *efg1*Δ/Δ, *efg1*Δ/Δ*set3*Δ/Δ and *efg1*Δ/Δ*hos2*Δ/Δ cultures neither in the white nor in the opaque phase. It is to note that we analysed the double deletion strains, because we repeatedly failed to maintain the *set3*Δ/Δ and *hos2*Δ/Δ single deletion mutants in relatively pure opaque phase cultures, most likely because they show an elevated opaque to white switching frequency (Table 1). However, because of the epistatic relationship of *SET3*, *HOS2* and *EFG1*, the input of the Set3/Hos2 pathway can be measured in an *EFG1*-deletion background, and the *efg1*Δ/Δ*set3*Δ/Δ as well as the *efg1*Δ/Δ*hos2*Δ/Δ mutants are marginally more stable in the opaque phase than the *set3*Δ/Δ and *hos2*Δ/Δ mutants (compare Tables 1 and 2). To address whether the Set3/Hos2 complex acts at other possible loci, we analysed the transcript levels of *WOR2*, the cofactor of *WOR1*, in wild type, *efg1*Δ/Δ, *efg1*Δ/Δ*set3*Δ/Δ and *efg1*Δ/Δ*hos2*Δ/Δ mutants, but again found no significant differences between wild type and the deletion mutants in either of the phases. Taken together, these results demonstrate that deletion of *SET1* and loss of H3K4 methylation suppresses the deletion of *SET3* or *HOS2*, suggesting that the Set3/Hos2 complex acts through Set1 and thus requires H3K4 methylation for switching regulation.

**Table 3:** Loss of *SET3* or *HOS2* is epistatic to the deletion of *HDA1* or *RPD31*.

	White → opaque		Opaque → white	
Strain	Switch (%)	*n*	Switch (%)	*n*
wt	11.6 ± 4.5	560	21.8 ± 9.5	609
*hda1*Δ/Δ*set3*Δ/Δ	1.4 ± 0.8	1295	NA	–
*rpd31*Δ/Δ*set3*Δ/Δ	2.1 ± 0.9	1409	NA	–
*rpd31*Δ/Δ*hos2*Δ/Δ	0.7 ± 0.3	1479	NA	–

Quantitative white to opaque (left panel) and opaque to white (right panel) switching assays were performed with multiple homozygous deletion mutants. The percentages represent the fraction of colonies that showed an alteration of the original phenotype. All strains are *MTL***a**/**a** strains. The opaque to white switching frequencies were not scored because of the opaque-specific filamentation phenotype caused by the loss of *SET3* or *HOS2* (see text). Data are displayed as a mean ± SD as well as the total number of colonies scored in three independent experiments carried out with the same strain.

NA, not assayed.

### Adenine facilitates opaque to white switching depending on *SET3*

Environmental factors can modulate morphogenetic switching of *C. albicans*. For instance, opaque cells convert to the white phase at elevated temperatures (Rikkerink *et al*., 1988), whereas white cells of certain strains readily convert to the opaque phase under anaerobic conditions (Ramirez-Zavala *et al*., 2008). To address whether our newly identified modulators link environmental signals to white–opaque switching, we analysed switching frequencies of wild type and several single deletion strains under different conditions, including changes in temperature, CO_2_^–^ and nutrient concentrations. Strikingly, we found that supplementation of the routinely used Lee's medium with 100 μg ml^−1^ adenine increased the conversion frequency from the opaque to the white phase. In these experiments, cells were grown on source plates for 5 days at 25°C, resuspended in water, spread onto destination plates at a low density and cultivated for another 5 days at 25°C. The effect was most pronounced if both plates contained adenine (Table 5). This regulatory effect is also supported by the observation that our wild type background strain showed elevated opaque to white switching frequencies on SD plates when compared with Lee's medium (Tables 1 and 3). Furthermore, the *set3*Δ/Δ deletion mutant displayed no alteration in opaque to white switching frequency in the presence of adenine in the medium (Table 5). These results identify adenine as a novel environmental factor regulating morphogenetic switching of *C. albicans*, and demonstrate that a functional Set3/Hos2 pathway is required to transmit the relevant input signal in the transcriptional circuit underlying switching.

**Table 5:** Adenine stimulates opaque-white switching in wild type but not in *set3*Δ/Δ cells.

			Switch	
Strain	Source plate (adenine)	Destination plate (adenine)	%	*n*
wt opaque	−	−	2.2 ± 0.9	376
		+	5.8 ± 1.9	286
	+	−	7.7 ± 3.7	290
	+	+	14.1 ± 6.6	260
*set3*Δ/Δ opaque	−	−	30.2 ± 2.7	180
		+	31.7 ± 2.5	202
	+	−	31.7 ± 4.8	250
	+	+	32.3 ± 1.0	228

Quantitative opaque to white switching assays were performed with multiple homozygous deletion mutants. Cells were incubated for 5 days on the source plates containing either none (–) or 100 μg ml^−1^ adenine (+) and were spread onto the destination plates containing none (–) or 100 μg ml^−1^ adenine (+). The percentages represent the fraction of white phase colonies scored after 5 days' incubation period. All strains are *MTL***a**/**a** strains. Data are displayed as a mean ± SD as well as the total number of colonies scored in three independent experiments carried out with the same strain.

## Discussion

### A model of white–opaque switching in *C. albicans* including two regulatory layers

In this study, we used the phenomenon of white–opaque switching in *C. albicans* to analyse the mechanisms of heritable phenotypic variation in a eukaryotic unicellular pathogen. The white and opaque cell types of *C. albicans* represent different transcriptional states of cells containing otherwise identical genomes. Morphogenetic switching is thought to generate distinct cell variants with different capabilities to adapt to various host niches and/or host defences *in vivo*. Current models explain the regulation of switching by a transcription circuitry amplifying stochastic changes of the expression of one master transcription factor gene, *WOR1*. Here, we used a reverse genetic approach to decipher the role of chromatin-modifying enzymes in white–opaque switching. Based on our data, we propose a novel dual-layer network model for the regulation of morphogenetic switching in *C. albicans* (Fig. 3C).

### Transcriptional layer of regulation

The principle architecture of the transcriptional circuit has been described earlier (Zordan *et al*., 2007). In *MTL* heterozygous cells, *WOR1* is repressed by the *MTL***a**/α repressor. In *MTL* homozygous white cells, Wor1 levels are low because *EFG1* represses *WOR2*, a putative cofactor of *WOR1.* Once the level or activity of Wor1 reaches a threshold, cells convert to the opaque phase. Conversely, opaque cells maintain Wor1 at high levels requiring multiple positive feedback loops mediated by Wor1, including (i) autoregulation of the *WOR1* locus; (ii) through the activation of *WOR2*; (iii) through repressing *EFG1* directly and also indirectly by activating *CZF1*, a repressor of Efg1. In this model, *EFG1* and *WOR1* promote the white and opaque phases respectively (Zordan *et al*., 2007). However, our data demonstrate that cells lacking both *EFG1* and *WOR1* not only fail to express opaque-specific genes such as *OP4* and *SAP1,* but also express the white-specific *WH11* transcript like wild type white cells (Fig. 1B). Wor1 in wild type opaque cells therefore must repress certain white-specific genes such as *WH11*, irrespective of its function to repress *EFG1*. This argues that wild type white cells are in the white phase, primarily because they lack Wor1 and not because they express Efg1. Furthermore, we demonstrate that *EFG1* regulates cellular morphology downstream of *WOR1* (Fig. 1A), arguing that wild type opaque cells display an elongated cell morphology mainly because of Efg1 expression occurs at lower levels than in round-shaped wild type white cells (Fig. 1B), confirming a previously proposed idea (Srikantha *et al*., 2000).

### Chromatin-level layer of regulation

In addition to transcriptional regulation, we found that several chromatin-modifying enzymes strongly modulate white–opaque switching. Notably, deletions of genes in the transcriptional circuit appear to have a more severe effect on white–opaque switching (Zordan *et al*., 2007). We think that even an apparently subtle twofold change in switching frequencies must require substantial changes in transcription, because the circuitry inherently ‘buffers’ small fluctuations by multiple feedback loops. The newly identified genes fall into distinct functional categories based on the direction(s) of switching they modulate. This strongly suggests that chromatin modifications modulate the activity of the underlying transcriptional network at multiple branching points. Interestingly, the phenotype of chromatin-modifier deletions in some cases correlates with phenotypes of transcription factor deletions, suggesting that they function at the same branch of the transcriptional circuit. For example, lack of *HST2* specifically results in a 10-fold decrease in the white to opaque switching, but fails to impact the opaque to white switching. Moreover, loss of *HST2* is suppressed by the loss of *EFG1* (Tables 1 and 2), while all of these effects are phenocopied by the deletion of *CZF1* (Zordan *et al*., 2007), indicating that *HST2* impacts transcriptional regulation at the *CZF1*-branch. This input could be, for instance, exerted either at the *CZF1* locus or through the *CZF1*-dependent repression of *EFG1*. In addition, loss of *NAT4* promotes the white phase, which is suppressed by the deletion of *EFG1* (Tables 1 and 2), suggesting that *NAT4* influences transcriptional activity at the *EFG1* locus, independent of *CZF1* (Fig. 3B). The formal possibility that *NAT4* modulates binding of Wor1 at the *EFG1* promoter seems plausible but requires further experimental confirmation.

Likewise, as loss of either *HOS2* or *SET3* promotes the white phase and their deletions suppress the loss of *EFG1*, both *HOS2* and *SET3* map to a pathway operating downstream of *EFG1* and upstream of *WOR1*, possibly at the *WOR1* or *WOR2* loci. Nevertheless, it may not be surprising that we did not observe differences in mRNA levels of either *WOR1* or *WOR2* in deletion mutants of the Set3/Hos2 pathway, because in *S. cerevisiae* loss of Hos2 was shown to change the transcription kinetics rather than the steady state transcript level of its target genes (Wang *et al*., 2002). Therefore, more direct experiments are needed to prove at which loci the Set3/Hos2 complex exerts its function to regulate white–opaque switching in *C. albicans*. Theoretically, the possibility that *SET3* or *HOS2* effects are exerted at the *WOR2* locus seems more unlikely, because overexpression of *WOR2* has no influence on switching rates (Zordan *et al*., 2007). Hence, the genetic circuitry appears relatively well buffered against fluctuations of *WOR2* levels.

### Set3 and Hos2 function as a complex in *C. albicans*

In this study, we provide four lines of genetic evidence that Set3 and Hos2 act as a complex in *C. albicans*. First, the phenotype of single deletions is identical; second, a loss of either *HOS2* or *SET3* is epistatic to the loss of *EFG1*; third, the loss of either *HOS2* or *SET3* is epistatic to the loss of *RPD31*; and fourth, deletion of *SET1* suppresses the loss of either *HOS2* or *SET3.* Hence, the situation in *C. albicans* appears similar to the Set3/Hos2 complex in *S. cerevisiae*, where deletion of either *SET3* or *HOS2* prevents assembly of a functional histone deacetylase complex *in vivo* (Pijnappel *et al*., 2001). Notably, a similar architecture is present in the mammalian HDAC3/SMRT complex, indicating a strong evolutionary conservation (Guenther *et al*., 2000).

Interestingly, loss of H3K4 methylation suppresses the disruption of the complex, suggesting that proper localization of Set3/Hos2 requires an interaction of the Set3 PHD finger with a methylated H3K4 residue. This notion is further supported by the finding that the purified PHD domain of ScSet3 specifically binds trimethylated H3K4 *in vitro* (Shi *et al*., 2007). In the context of our work, it is important to note that loss of *SET1* failed to revert the opaque filamentation phenotype of the *hos2*Δ/Δ and *set3*Δ/Δ mutants. This strongly suggests that Set3/Hos2 localization requires H3K4 methylation only at specific loci. An alternative way to interpret the epistasis relationships of *SET1*, *SET3* and *HOS2* is that *SET1* and methylation of H3K4 represses white-to-opaque switching. The Set3/Hos2 complex counteracts this repressive effect. Consequently, in *set1*Δ/Δ mutant cells lacking methylation of H3K4, Set3/Hos2 seems dispensable for establishing a normal white-to-opaque switching rate. Further biochemical assays with appropriate tools which are currently being developed will be necessary to establish detailed mechanistic relationships and the interplay of these gene products. However, we provide compelling genetic evidence that *SET1*, *SET3* and *HOS2* define a novel pathway regulating *WOR1* expression and white–opaque switching in *C. albicans*.

### Environmental control of phenotypic switching

Although white–opaque switching is a unique characteristic of *C. albicans*, reversible switching between distinct phenotypes has been described in a vast number of microbes, including the non-pathogenic yeast *S. cerevisiae*, the pathogen *Cryptococcus neoformans*, the protozoan parasites *Trypanosoma brucei* and *Plasmodium falciparum,* as well as many prokaryotic microbes such as *Escherichia coli* and *Bacillus subtilis* (Avery, 2006)*.* Typically, two extreme cases of switching are considered: responsive switching occurs as a direct consequence of a change in environmental conditions, which is sensed by a dedicated apparatus; alternatively, stochastic switching occurs without an outside input mostly as a result of intrinsic transcriptional fluctuations of one or more regulatory genes (Kaern *et al*., 2005; Kussell and Leibler, 2005). Theoretical models support that stochastic switching of phenotypes is favourable when the environment only seldom changes. By contrast, the more fluctuating the environment is, the more beneficial it is to have a sensing apparatus enabling the cells to actively respond to changes (Kussell and Leibler, 2005).

Recent models explain white–opaque switching as a stochastic process, whereby the fluctuations of one central factor (*WOR1*), along with at least three other regulators (*WOR2*, *CZF1*, *EFG1*), are buffered by multiple feedback loops (Huang *et al*., 2006; Zordan *et al*., 2006; 2007). On the other hand, several lines of evidence in the literature and data presented here argue that white–opaque switching is likely to respond to environmental or even host stimuli. For instance, high temperature causes opaque cells to convert *en masse* to the white phase (Rikkerink *et al*., 1988). Conversely, anaerobic conditions in some strains promote the formation of opaque cells both *in vitro* and more significantly, in the murine gastrointestinal tract (Ramirez-Zavala *et al*., 2008). Most notably, the latter study also revealed that *CZF1* is required for the anaerobiosis-induced white-to-opaque conversion in the strain WO-1, which interlinks environmental sensing and the genetic circuit driving white–opaque switching. The notion that the regulation of white–opaque switching is likely to have a responsive nature is further supported by numerous studies that report differences of white and opaque cells in their abilities to adapt to various host niches. It is fair to state that infectious microbes encounter many distinct local environments of varying parameters during an infection of the human host. Opaque phase *C. albicans* cells, for instance, are better colonizers of the skin, whereas white phase cells are more prevalent in bloodstream infections (Kvaal *et al*., 1999). Furthermore, opaque cells are more susceptible to killing by neutrophils than white cells (Kolotila and Diamond, 1990), whereas macrophages preferentially phagocytose white cells over opaque cells (Lohse and Johnson, 2008).

The cell-fate decision machinery may encounter many different sometimes conflicting signals, which need proper processing in order to adapt in a favourable fashion. We propose that changing the chromatin status at adequate regulatory loci is a plausible mechanism to integrate multiple environmental stimuli. Notably, although an elevated temperature and anaerobiosis favour the white and the opaque phenotype respectively, anaerobic conditions stabilize opaque cells even at elevated temperatures *in vitro* (Dumitru *et al*., 2007). Furthermore, white cells of some strains convert to the opaque phase in the murine gastrointestinal tract, whose 37°C temperature is much higher than 25°C, the normal laboratory condition used to stably propagate opaque cells (Ramirez-Zavala *et al*., 2008). Such a proposed relay function of chromatin is further supported by the finding that Wor1 in opaque cells can be immunoprecipitated from promoters of many genes whose expression does not change during the white–opaque switch under laboratory conditions (Zordan *et al*., 2007).

In this study, we identify another novel external stimulus, showing that the presence of adenine facilitates opaque to white switching *in vitro*, and, importantly, that *SET3* is required for this regulatory effect. This finding is in very good agreement with the proposed functional consequence of the dual layer model, whereby the chromatin modifiers are involved in the integration of environmental stimuli to shape cell fate. Notably, it was recently shown that the nicotinic acid (a precursor for NAD) concentration of urine regulates the adherence properties of the related species *Candida glabrata* in a urinary tract infection model, and that chromatin-mediated gene silencing is linked to the process (Domergue *et al*., 2005). Therefore, it will be interesting in the future to test whether nucleotide or nucleotide precursor concentrations and chromatin-based regulatory mechanisms also play a role in *C. albicans* infection models *in vivo*.

The mechanism(s) driving morphogenetic switching thus may be quite simple. For instance, changes in the chromatin modification status could directly or indirectly fine-tune promoter occupancy of transcription factors by changing their affinities or modulate the assembly of the mediator complex. Alternatively, it is tantalizing to speculate that histone modifiers may even cause changes in post-translational modifications of transcription factors by modulating complex assembly.

Finally, *C. albicans* during its commensalistic co-evolution with the human host must have developed elaborate systems of specific and rapidly acting sensing mechanisms to allow for environmental and host signal integration. This machinery consists of two layers: a transcriptional level which co-ordinates the downstream response at the gene expression level, and a chromatin-level layer that may have a relay function at key loci integrating the stimuli affecting cellular identity. Moreover, the architecture combining specific transcription factors with chromatin modifiers is reminiscent of the cell-fate and developmental decision machineries in higher eukaryotic systems. Indeed, a differential chromatin status at key loci has been linked to lineage-committed stem cell differentiation (Mikkelsen *et al*., 2007). Strikingly, a selective H3K4 methylation pattern has been recently linked to lineage commitment during hematopoesis (Orford *et al*., 2008). These and other similarities make *C. albicans* an attractive alternative model system to dissect the molecular mechanisms of chromatin dynamics and enzyme recruitment to delineate developmental processes controlling cell-fate decisions and developmental changes.

## Experimental procedures

### Media and growth conditions

Rich medium (YPD) and complete synthetic medium (SD) was prepared as previously described (Kaiser *et al*., 1994). Modified Lee's medium was prepared as described (Bedell and Soll, 1979). Cultures were routinely grown at 25°C unless indicated otherwise.

### Strain construction

The complete list of *C. albicans* strains, primers and plasmids used in this study are listed in Tables S2, S3 and S4 respectively. All strains were derived from SN152 (Noble and Johnson, 2005), a leucine, histidine, arginine auxotrophic derivative of the clinical isolate SC5314 (Gillum *et al*., 1984). The *MTL***a**/α SN152 was cultured on sorbose medium (Janbon *et al*., 1998) to construct the *MTL***a**/**a** strain DHCA202. *MTL* homozygosis was verified by PCR and Southern blot analyses (data not shown). Single gene deletions (*SET1*, *SET2*, *HDA1*, *SAS2*, *RPD31*, *SET3*, *HOS2*, *HST1*, *SIR2*, *HST2*, *ELP3*, *PHO13*, *PHO8*, *DOT1*, *HOS1*, *HPA2*, *HOS3* and *WOR1*) were created by using the *C.m.LEU2* and *C.d.HIS1* marker cassettes as described in Noble and Johnson (2005). In addition, the same strategy utilizing the *C.d.ARG4* and a *SAT1* cassette (amplified from the plasmid pSFS2A) was used to delete *WOR1* in the *set1*Δ/Δ, *hos2*Δ/Δ, *set3*Δ/Δ and *set1*Δ/Δ*hos2*Δ/Δ backgrounds.

Other multiple gene deletion mutants, as well as the *efg1*Δ/Δ in the DHCA202 and SC5314 backgrounds were created using the ‘*SAT1*-flipping’ method (Reuss *et al*., 2004). *EFG1* was deleted in the *wor1*Δ/Δ, *hos2*Δ/Δ, *set3*Δ/Δ, *hst2*Δ/Δ, *nat4*Δ/Δ and *set1*Δ/Δ single deletion strains to create all possible double deletions. Likewise, *SET3* and *HOS2* were deleted in the single deletion strains *hda1*Δ/Δ, *rpd31*Δ/Δ, *hst2*Δ/Δ, *nat4*Δ/Δ or *set1*Δ/Δ to obtain all double mutants. Moreover, *EFG1* was deleted in the *hst2*Δ/Δ*hos2*Δ/Δ, *nat4*Δ/Δ*set3*Δ/Δ and *set1*Δ/Δ*hos2*Δ/Δ double deletion backgrounds to construct the corresponding triple deletion strains. Except for single gene deletions that did not display any phenotypes (Table S2), at least two independent homozygous deletion strains were created derived from independent heterozygote isolates. Transformation was performed via electroporation as described (Reuss *et al*., 2004). Genomic integration events were verified with PCR and Southern blot analyses (data not shown). The mating tester strains DHCA210 (*MTL*α/α) and DHCA209 (*MTL***a**/**a**) were created in the SC5314 background using the sorbose selection method. Subsequent disruption of the *ADE2* gene used the ‘SAT1-flipping’ strategy (see Tables S2 and S3).

Gene complementation constructs for the *HOS2*, *HST2* and *NAT4* ORFs were created using the *SAT1* marker cassette of the plasmid pSFS2A and the fusion PCR strategy (Noble and Johnson, 2005). For the restoration of the *SET3* gene, the *SAT1*-flipping strategy was used with the modification that the in pSFS2A plasmid the upstream homology region was replaced by the same upstream region and the coding sequence (Tables S3 and S4). Transformation was performed via electroporation as described (Reuss *et al*., 2004). Genomic integration events were verified with PCR analysis.

### Microscopy

Colony morphology was analysed using a Discovery V12 Stereoscope (Zeiss) equipped with an Axiocam MR5 camera (Zeiss). Microscopic analysis was performed with using an Axioplan 2 microscope (Zeiss) equipped with a Spot Pursuit camera (Sony). Images were analysed with the Axiovision 4.1 software (Zeiss).

### White–opaque switching assays

Quantitative switching assays were performed as previously described (Miller and Johnson, 2002) with modifications. Briefly, white strains were streaked from frozen stocks on YPD plates and grown at 30°C for 2 days. Single colonies were then restreaked onto modified Lee's medium (Tables 1 and 2) or SD medium (Tables 3 and 4) and grown at 25°C for 5 days. Single colonies were picked and resuspended in sterile H_2_O, checked by microscopy and spread onto modified Lee's plates (Tables 1 and 2) or SD plates (Tables 3 and 4) containing 5 μg ml^−1^ Phloxin B. Formation of opaque colonies or sectors was scored after 7 days. The opaque to white switching assays were performed using pure opaque colonies obtained in the white to opaque switching assays. The frequency of white colonies or colonies containing at least one white sector was scored after 7 days. For each strain, at least three independent experiments were carried out. The data listed in Tables 1–5 were obtained using one deletion strain of the genotype. For each genotype except for the *set1*Δ/Δ*set3*Δ/Δ, *wor1*Δ/Δ*hos2*Δ/Δ and *wor1*Δ/Δ*set1*Δ/Δ*hos2*Δ/Δ mutants at least two independent homozygous deletion strains were created derived from independent heterozygous deletion strains. The analysis of independent deletion mutants showed qualitatively similar results (data not shown).

### Quantitative mating assays

Quantitative mating assays were performed essentially as described (Miller and Johnson, 2002) with modifications. Pure white and opaque cultures were isolated on plates as described above. Strains were grown in liquid medium at 25°C until an OD_600_ 1–3. A total of 3 × 10^7^ cells of each mating partner were mixed, and deposited on sterile Whatman filter paper placed onto a YPD plate supplemented with 100 μg ml^−1^ adenine, and incubated at 25°C for 18 h. Cells were washed off the filter, resuspended in 10 ml sterile H_2_O and were dispersed by vortex-mixing. Serial dilutions were plated on double-selective (–arginine –adenine) SD plates to select for the prototrophic conjugants, and on single selective (–arginine or –adenine) SD plates to score the single parent population plus conjugants. The mating frequencies were calculated as the ratio of conjugants and the limiting parent plus conjugants.

### RNA isolation and quantitative RT-PCR

Cultures were grown in modified Lee's medium until OD_600_ 1–3 and harvested by centrifugation. Pellets were washed with sterile H_2_O, frozen in liquid nitrogen and mechanically pulverized in a sterile porcelain mortar in the frozen state. RNA was extracted using TRI reagent (Molecular Research Center). About 1–5 μg of total RNA was reverse-transcribed with the First Strand cDNA synthesis kit (Fermentas). cDNA amplification was monitored quantitatively by SYBR Green incorporation in a Realplex Mastercycler (Eppendorf).

### Immunoblotting

Cultures were grown in liquid medium until OD 1–3 and cells were harvested by centrifugation. Cell pellets were resuspended in 0.25 M NaOH and 1% β-mercaptoethanol, and incubated on ice for 10 min. Proteins were precipitated by the addition of 5.8 v/v% trichloroacetic acid for 10 min on ice, centrifuged and resuspended in SDS sample buffer. Total protein extracts derived from 0.5 OD_600_ of the starting cultures were separated by SDS/PAGE and analysed by Western blotting. The C-terminal anti-Wor1 antibody has been previously described (Zordan *et al*., 2006). Loading controls were visualized using a monoclonal anti-tubulin antibody (Sigma).
