# Supplementary material for: Transcriptional loops meet chromatin: a dual-layer network controls white–opaque switching in Candida albicans
Source: Mol Microbiol. 2009 Jul 6;74(1):1–15. doi: 10.1111/j.1365-2958.2009.06772.x (PMC2764112; doi:10.1111/j.1365-2958.2009.06772.x)
Supplement: Supplementary file 1 [file mmi0074-0001-SD1.pdf]

Supplementary Material

Supplementary Table 1. Open Reading Frames (ORFs) identified as putative switching modulators

Function	*S. cerevisiae* Gene Name	*C. albicans* ORF	Protein Length	Protein BLAST E-value**	*C. albicans* Deletion(s) created***	*S. cerevisiae* Reference	*C.albicans* Reference
**HMT** (KMT)§	*SET1(KMT2)*§	19.6009	1040	$4 \cdot 10^{-101}$	+ +	(Roguev *et al.*, 2001)	(Raman *et al.*, 2006)
	SET2(KMT3)§	19.1755	844	$1 \cdot 10^{-115}$	+		
	DOT1(KMT4)§	19.7402	1343	$2 \cdot 10^{-56}$	+		
**HDPH**	*PHO8*	19.4736	551	$<10^{-150}$	+		
	PHO13	19.4444	308	$5 \cdot 10^{-78}$	+ +	(Tuleva *et al.*, 1998)	
**HAT** (KAT)§	*GCN5(KAT2)*§	19.705	449	$<10^{-150}$			
	SAS2(KAT8)§	19.2087	352	$2 \cdot 10^{-60}$	+		
	HAT1(KAT1)§	19.779	413	$7 \cdot 10^{-82}$			
	SAS3(KAT6)§	19.2540	805	$8 \cdot 10^{-98}$			
	ELP3(KAT9)§	19.7387	547	$<10^{-150}$	+		
	NAT4	19.4664	226	$3 \cdot 10^{-13}$	+ +	(Song *et al.*, 2003)	
	SPT10	19.2361	469	$3 \cdot 10^{-60}$	+		
	HPA2	19.6323	192	$5 \cdot 10^{-32}$	+		
**HDAC**	*HDA1*	19.2606	833	$<10^{-150}$	+ +	(Carmen *et al.*, 1996)	(Klar *et al.*, 2001)
	RPD3	19.6801 *(RPD3)*	577	$<10^{-150}$		(Rundlett *et al.*, 1996)	(Srikantha *et al.*, 2001)
	RPD3	19.2834 *(RPD31)*	480	$<10^{-150}$	+ +		
	SIR2	19.1992	515	$4 \cdot 10^{-103}$	+		
	HST1	19.4761	657	$5 \cdot 10^{-105}$	+ +	(Xie *et al.*, 1999)	
	HST2	19.2580	331	$2 \cdot 10^{-72}$	+ +	(Landry *et al.*, 2000)	
	HOS1	19.4411	436	$2 \cdot 10^{-56}$	+		

	HOS2	19.5377	454	$<10^{-150}$	+ +	(Pijnappel *et al.*, 2001)	
	HOS3	19.2772	713	$<10^{-150}$	+		
**Additional**[*]	*SET3*	19.7221	1069	$2·10^{-47}$	+ +	(Pijnappel et al., 2001)	

HMT: histone methyltransferase, HDPH: histone dephosphorlyase, HAT: histone acetyltransferase, HDAC: histone deacetylase, KMT: K-Methyltransferase, KAT: K-Acetyltransferase

[*] HDAC complex subunit; [**] Performed at http://www.ncbi.nlm.nih.gov/blast/Blast.cgi using the algorithm described in (Altschul *et al.*, 1990)

[***] +: One mutant of the genotype was created and analyzed (with no effect found on white-opaque switching regulation), ++: at least two independent mutants of the genotype was created and analyzed

[§] Name according to the nomenclature described in (Allis *et al.*, 2007)

Supplementary Table 2. *C. albicans* strains used in this study

Description	Name*	*MTL*	Parent	Genotype	Reference	Figure/Table
wt	SC5314	**a**/α	Clinical isolate		(Gillum *et al.*, 1984)	
wt	L26	**a**/**a**	Clinical isolate		(Lockhart *et al.*, 2002)	
wt	SN152	**a**/α	SC5314	*arg4Δ/arg4Δ his1Δ/his1Δ leu2Δ/leu2Δ URA3/ura3Δ::λimm*^{434} *IRO1/iro1Δ::λimm*^{434}	(Noble & Johnson, 2005)	Figure 1
wt	DHCA202	**a**/**a**	SN152	*arg4Δ/arg4Δ his1Δ/his1Δ leu2Δ/leu2Δ URA3/ura3Δ::λimm*^{434} *IRO1/iro1Δ::λimm*^{434}	This study	Figures 1, 2 Tables 1, 2, 3, 4, 5
efg1Δ/Δ	DHCA216	**a**/α	SC5314	*efg1Δ*::FRT/*efg1Δ*::FRT	This study	Figure 1
efg1Δ/Δ	HLC67	**a**/α	SC5314	*ura3Δ::λimm*^{434}*/ura3Δ::λimm*^{434} *efg1Δ::hisG/efg1Δ::hisG*	(Lo *et al.*, 1997)	Figure 1C
efg1Δ/Δ	DHCA212 DHCA214	**a**/**a**	DHCA202	*efg1Δ*::FRT/*efg1Δ*::FRT	This study	Figure 1, Table 3
wor1Δ/Δ	DHCA218 DHCA220	**a**/**a**	DHCA202	*wor1Δ::C.d.HIS1/wor1Δ::C.m.LEU2*	This study (Zordan *et al.*, 2006)	Figure 1
efg1Δ/Δ wor1Δ/Δ	DHCA222 DHCA224	**a**/**a**	DHCA202	*efg1Δ*::FRT/*efg1Δ*::FRT *wor1Δ::C.d.HIS1/wor1Δ::C.m.LEU2*	This study	Figure 1
set1Δ/Δ	DHCA226 DHCA228	**a**/**a**	DHCA202	*set1Δ::C.d.HIS1/set1Δ::C.m.LEU2*	This study	Table 1, Figure 2
hda1Δ/Δ	DHCA232 DHCA234	**a**/**a**	DHCA202	*hda1Δ::C.d.HIS1/hda1Δ::C.m.LEU2*	This study	Table 1, Figure 2
rpd31Δ/Δ	DHCA238 DHCA240	**a**/**a**	DHCA202	*rpd31Δ::C.d.HIS1/rpd31Δ::C.m.LEU2*	This study	Table 1, Figure 2
set3Δ/Δ	DHCA242 DHCA244	**a**/**a**	DHCA202	*set3Δ::C.d.HIS1/set3Δ::C.m.LEU2*	This study	Table 1, Table 5 Figure 2
hos2Δ/Δ	DHCA246 DHCA248	**a**/**a**	DHCA202	*hos2Δ::C.d.HIS1/hos2Δ::C.m.LEU2*	This study	Table 1, Figure 2
hst1Δ/Δ	DHCA250 DHCA252	**a**/**a**	DHCA202	*hst1Δ::C.d.HIS1/hst1Δ::C.m.LEU2*	This study	Table 1, Figure 2
hst2Δ/Δ	DHCA256 DHCA258	**a**/**a**	DHCA202	*hst2Δ::C.d.HIS1/hst2Δ::C.m.LEU2*	This study	Table 1, Figure 2
pho13Δ/Δ	DHCA262 DHCA264	**a**/**a**	DHCA202	*pho13Δ::C.d.HIS1/pho13Δ::C.m.LEU2*	This study	Table 1, Figure 2
nat4Δ/Δ	DHCA268 DHCA270	**a**/**a**	DHCA202	*nat4Δ::C.d.HIS1/nat4Δ::C.m.LEU2*	This study	Table 1, Figure 2
efg1Δ/Δ hos2Δ/Δ	DHCA302 DHCA304	**a**/**a**	DHCA202	*hos2Δ::C.d.HIS1/hos2Δ::C.m.LEU2* *efg1Δ*::FRT/*efg1Δ*::FRT	This study	Table 2

efg1Δ/Δ set3Δ/Δ	DHCA298 DHCA300	**a/a**	DHCA202	set3Δ::C.d.HIS1/set3Δ::C.m.LEU2<br>efg1Δ::FRT/efg1Δ::FRT	This study	Table 2
efg1Δ/Δ hst2Δ/Δ	DHCA306 DHCA308	**a/a**	DHCA202	hst2Δ::C.d.HIS1/hst2Δ::C.m.LEU2<br>efg1Δ::FRT/efg1Δ::FRT	This study	Table 2
efg1Δ/Δ nat4Δ/Δ	DHCA310 DHCA312	**a/a**	DHCA202	nat4Δ::C.d.HIS1/nat4Δ::C.m.LEU2<br>efg1Δ::FRT/efg1Δ::FRT	This study	Table 2
efg1Δ/Δ hst2Δ/Δ<br>hos2Δ/Δ	DHCA318 DHCA320	**a/a**	DHCA202	hst2Δ::C.d.HIS1/hst2Δ::C.m.LEU2<br>hos2Δ::FRT/hos2Δ::FRT<br>efg1Δ::FRT/efg1Δ::FRT	This study	Table 2
efg1Δ/Δ nat4Δ/Δ<br>set3Δ/Δ	DHCA322 DHCA324	**a/a**	DHCA202	nat4Δ::C.d.HIS1/nat4Δ::C.m.LEU2<br>set3Δ::FRT/set3Δ::FRT<br>efg1Δ::FRT/efg1Δ::FRT	This study	Table 2
rpd31Δ/Δ hos2Δ/Δ	DHCA290 DHCA292	**a/a**	DHCA202	rpd31Δ::C.d.HIS1/rpd31Δ::C.m.LEU2<br>hos2Δ::FRT/hos2Δ::FRT	This study	Table 3
hda1Δ/Δ set3Δ/Δ	DHCA282 DHCA284	**a/a**	DHCA202	hda1Δ::C.d.HIS1/hda1Δ::C.m.LEU2<br>set3Δ::FRT/set3Δ::FRT	This study	Table 3
rpd31Δ/Δ set3Δ/Δ	DHCA286 DHCA288	**a/a**	DHCA202	rpd31Δ::C.d.HIS1/rpd31Δ::C.m.LEU2<br>set3Δ::FRT/set3Δ::FRT	This study	Table 3
set1Δ/Δ hos2Δ/Δ	DHCA326 DHCA328	**a/a**	DHCA202	set1Δ::C.d.HIS1/set1Δ::C.m.LEU2<br>hos2Δ::FRT/hos2Δ::FRT	This study	Table 4
set1Δ/Δ set3Δ/Δ	DHCA330	**a/a**	DHCA202	set1Δ::C.d.HIS1/set1Δ::C.m.LEU2<br>set3Δ::FRT/set3Δ::FRT	This study	Table 4
efg1Δ/Δ set1Δ/Δ	DHCA334 DHCA336	**a/a**	DHCA202	set1Δ::C.d.HIS1/set1Δ::C.m.LEU2<br>efg1Δ:FRT/efg1Δ:FRT	This study	Table 4
efg1Δ/Δ set1Δ/Δ<br>hos2Δ/Δ	DHCA338 DHCA340	**a/a**	DHCA202	set1Δ::C.d.HIS1/set1Δ::C.m.LEU2<br>hos2Δ::FRT/hos2Δ::FRT<br>efg1Δ::FRT/efg1Δ::FRT	This study	Table 4
set1Δ/Δ wor1Δ/Δ	DHCA342 DHCA344	**a/a**	DHCA202	set1Δ::C.d.HIS1/set1Δ::C.m.LEU2<br>wor1Δ::C.d.ARG4/wor1Δ::SAT1	This study	Table 4
set3Δ/Δ wor1Δ/Δ	DHCA346 DHCA348	**a/a**	DHCA202	set3Δ::C.d.HIS1/set3Δ::C.m.LEU2<br>wor1Δ::C.d.ARG4/wor1Δ::SAT1	This study	Table 4
hos2Δ/Δ wor1Δ/Δ	DHCA350	**a/a**	DHCA202	hos2Δ::C.d.HIS1/hos2Δ::C.m.LEU2<br>wor1Δ::C.d.ARG4/wor1Δ::SAT1	This study	Table 4
set1Δ/Δ hos2Δ/Δ<br>wor1Δ/Δ	DHCA352	**a/a**	DHCA202	set1Δ::C.d.HIS1/set1Δ::C.m.LEU2<br>hos2Δ::FRT/hos2Δ::FRT<br>wor1Δ::C.d.ARG4/wor1Δ::SAT1	This study	Table 4

**Gene complementation mutants**						
*set3*Δ/*SET3*	DHCA353	**a**/**a**	DHCA202	*set3*Δ::*C.m.LEU2/SET3*::FRT	This study	
*hos2*Δ/*HOS2*	DHCA354	**a**/**a**	DHCA202	*hos2*Δ::*C.d.HIS1/HOS2*::*SAT1*	This study	
*hst2*Δ/*HST2*	DHCA355	**a**/**a**	DHCA202	*hst2*Δ::*C.m.LEU2/HST2*::*SAT1*	This study	
*nat4*Δ/*NAT4*	DHCA356	**a**/**a**	DHCA202	*nat4*Δ::*C.d.HIS1/NAT4::SAT1*	This study	
*efg1*Δ/Δ *set3*Δ/*SET3*	DHCA357	**a**/**a**	DHCA202	*set3*Δ::*C.d.HIS1/ SET3*::FRT *efg1*Δ::FRT/*efg1*Δ::FRT	This study	
*efg1*Δ/Δ *hos2*Δ/*HOS2*	DHCA358	**a**/**a**	DHCA202	*hos2*Δ::*C.m.LEU2/HOS2*::*SAT1* *efg1*Δ::FRT/*efg1*Δ::FRT	This study	
**Mating tester strains**						
Maa (mating tester)	DHCA209	**a**/**a**	SC5314	*ade2*Δ::FRT/*ade2*Δ::FRT	This study	Figure 1C
Mαα (mating tester)	DHCA210	α/α	SC5314	*ade2*Δ::FRT/*ade2*Δ::FRT	This study	Figures 1C, 2B
**Additional mutants**						
*set2*Δ/Δ	DHCA230	**a**/**a**	DHCA202	*set2*Δ::*C.d.HIS1*/*set2*Δ::*C.m.LEU2*	This study	
*sir2*Δ/Δ	DHCA254	**a**/**a**	DHCA202	*sir2*Δ::*C.d.HIS1*/*sir2*Δ::*C.m.LEU2*	This study	
*elp3*Δ/Δ	DHCA260	**a**/**a**	DHCA202	*elp3*Δ::*C.d.HIS1*/*elp3*Δ::*C.m.LEU2*	This study	
*pho8*Δ/Δ	DHCA266	**a**/**a**	DHCA202	*pho8*Δ::*C.d.HIS1*/*pho8*Δ::*C.m.LEU2*	This study	
*dot1*Δ/Δ	DHCA272	**a**/**a**	DHCA202	*dot1*Δ::*C.d.HIS1*/*dot1*Δ::*C.m.LEU2*	This study	
*hos1*Δ/Δ	DHCA274	**a**/**a**	DHCA202	*hos1*Δ::*C.d.HIS1*/*hos1*Δ::*C.m.LEU2*	This study	
*hos3*Δ/Δ	DHCA280	**a**/**a**	DHCA202	*hos3*Δ::*C.d.HIS1*/*hos3*Δ::*C.m.LEU2*	This study	
*spt10*Δ/Δ	DHCA276	**a**/**a**	DHCA202	*spt10*Δ::*C.d.HIS1*/*spt10*Δ::*C.m.LEU2*	This study	
*hpa2*Δ/Δ	DHCA278	**a**/**a**	DHCA202	*hpa2*Δ::*C.d.HIS1*/*hpa2*Δ::*C.m.LEU2*	This study	

*In case of multiple deletion mutants per genotype, the data displayed in Figure 1and 2, Tables 1, 2, 3 and 4 were derived from assays performed with the mutant of the lower index number (as described in the Experimental Procedures).

Supplementary Table 3. Oligonucleotide primers used in this study

Name	Sequence (5´- 3´)*	Reference
**Gene deletion constructs based on fusion PCR strategy** (Noble & Johnson, 2005)		
5C_CA6009	CGGTTTTCACTTAACTGCTGC	
53_CA6009	cacggcgcgcctagcagcggCATAGCAAGGTGTATGTTTGATTAC	
35_CA6009	gtcagcggccgcatccctgcTAATTGCATAAACGTGTGATAAATC	
33_CA6009	TTAGTAGTAGGCGAATAGACACAGAC	
55_CA1755	GATCTTGAAGAAGTAATGTTTCCTG	
53_CA1755	cacggcgcgcctagcagcggCATGATTTGTTAAATGAGTGTTTATC	
35_CA1755	gtcagcggccgcatccctgcTAGATATTTATTTTGTTCATACAAGAAAAA	
33_CA1755	GTTTCTGCTGAAGAAGCCG	
55_CA2606	GTGTGAGTAGAGTTCACAACAAGTG	
53_CA2606	cacggcgcgcctagcagcggCATTCTTAAAAAGGAAAATGATAATAG	
35_CA2606	gtcagcggccgcatccctgcTGATTCGAGTAGAAACAACAACAAC	
33_CA2606	CAACACTTTCCAAACACTCTCAG	
55_CA2087	AAGGCAAGAAGAGAATCTTTGG	
53_CA2087	cacggcgcgcctagcagcggCATGCATGAACTTTTATGATATTAGATG	
35_CA2087	gtcagcggccgcatccctgcTGAATTAGTTACATACTTTTTTTTTTTAAA	
33_CA2087	ACATGGAATACGTTGACTGGG	
55_CA6801	AATAACAGTTACTGTCACCGCC	
53_CA6801	cacggcgcgcctagcagcggCATGGTGGACGAGTTTGGTTG	
35_CA6801	gtcagcggccgcatccctgcTAAAAGTTCATAAATAAAAGGATATTTAGATTG	
33_CA6801	TCCGAAGGGAATTTAATTGG	
55_CA7221	ATTGTACGAAGAAGCGGAGC	
53_CA7221	cacggcgcgcctagcagcggCATTAAAATAAACACTTATAAAGACTACTATC	
35_CA7221	gtcagcggccgcatccctgcTAGTTTTTTGTTTAGAGTTTGTATATTG	
33_CA7221	CAAAAGGACAATCAATTGGATG	
55_CA5377	CAGAATCTTGACCTGTGATTCC	
53_CA5377	cacggcgcgcctagcagcggCATTTATATTAACTACTTTTCTCCTATGG	
35_CA5377	gtcagcggccgcatccctgcTAGTTTGTCTTGATACACATATACATATATATATA	
33_CA5377	GAAAAATGGATGCCAAGTTG	
55_CA4761	CACCCTTTCCTCTATTCTTTGC	
53_CA4761	cacggcgcgcctagcagcggCATTATTGATTGTTATTTGTTATTAGTAATAATTG	
35_CA4761	gtcagcggccgcatccctgcTGAAGAGCCAAAACAAGATAAAG	
33_CA4761	TATTGCTGCTTATAATGTATAGGGAG	

55_CA1992	ATCACAACAGTAGGGCATCAAC	
53_CA1992	cacggcgcgcctagcagcggCATTACTAGAGGATTTCTCTCAAATAAC	
35_CA1992	gtcagcggccgcatccctgcTGATTAAATTAATATTGGTGTCTTTAATG	
33_CA1992	TCAAGATCAACAATATGTGGTGG	
55_CA2580	CATTCGAGCTAAACGAAGCTC	
53_CA2580	cacggcgcgcctagcagcggCATGGTTTAAAGTTATAGTCTGTTGTAAG	
35_CA2580	gtcagcggccgcatccctgcTTAAACTGTCTCAACAAAAATATAATATAC	
33_CA2580	GCCTTCATAAAACGTTCTCTCAC	
55_CA7387	AAGTCTGACATTACACCAATATAAGG	
53_CA7387	cacggcgcgcctagcagcggCATTATGTAGTGTGTTTATTAGGAGAAG	
35_CA7387	gtcagcggccgcatccctgcTAAGACTTGATACTTACAGATTTGTATATG	
33_CA7387	CTATTGTTGATGGTGAACCACC	
55_CA4444	AGATAAAGAGTTGTTCTCTCAGTTCC	
53_CA4444	cacggcgcgcctagcagcggCATTAGAAACGGAATGTATTGGG	
35_CA4444	gtcagcggccgcatccctgcTAGAAACATATGCCTTGCTATTTTT	
33_CA4444	TGCAAGCAAAACTAAGAACGTC	
55_CA4736	CCTATCTTTACACTACTGTTCACCG	
53_CA4736	cacggcgcgcctagcagcggCATGATGGAATATGTAGCAATAAAAAG	
35_CA4736	gtcagcggccgcatccctgcTAAGTGTGTATGTACATATACCAAGAAC	
33_CA4736	ACAATGAAGCAAGTGAATAACG	
55_CA4664	TTTATTGGTTCTCAATGGCAG	
53_CA4664	cacggcgcgcctagcagcggCATTGTTGTTTGTGTTGGCA	
35_CA4664	gtcagcggccgcatccctgcTAAGTATTTGTGTAGAACATTCTTCCC	
33_CA4664	CAACAAATGGGTAGCAAGGTC	
55_CA7402	CGCTCCTAATTTCTCCAAGC	
53_CA7402	cacggcgcgcctagcagcggCATTATTAGTATTTGTCAAGTGCACAC	
35_CA7402	gtcagcggccgcatccctgcTAAAATGTTTTATAGCTAAGCTGTACC	
33_CA7402	AAATATGGCTTATCGACGGAG	
55_CA4411	TGCCAGTAAACGAGGTTACG	
53_CA4411	cacggcgcgcctagcagcggCATTAATCTATTAACAATAATACAAACCAG	
35_CA4411	gtcagcggccgcatccctgcTAAAATTATATATCTACCACTAAACTGTTTAAG	
33_CA4411	TTGTTACTGGAAAGGGACCC	
55_CA2361	TCTTCATCGTAATACGACGGTC	
53_CA2361	cacggcgcgcctagcagcggCATGATCTATTTAAAGATTGTAAGGAAT	
35_CA2361	gtcagcggccgcatccctgcTAGTTAAAAAGATCACACACTTACTGTTG	
33_CA2361	TGATTTCGATCAAAGAGCTGC	

55_CA6323	TGTTATGTATGAATGCACCGAG	
53_CA6323	cacggcgcgcctagcagcggCATTGACCCCTGGCTCCG	
35_CA6323	gtcagcggccgcatccctgcTGACCAATGTACACGACCTATG	
33_CA6323	TGAATCTTCAATTCATCGGC	
55_CA2772	CAATGGAAATATTGTTACAGATGG	
53_CA2772	cacggcgcgcctagcagcggCATTATAAAAAACTTCCCCAAGTAA	
35_CA2772	gtcagcggccgcatccctgcTAGAGTATATTAGAACATGAAATGTATATATATAAG	
33_CA2772	AACAACTACAACAACTACTACTACTCCAC	
55_CA4884	GCCTTTCCTGTATTGGTATTGG	
53_CA4884	cacggcgcgcctagcagcggCATTGCTTAATATTGAATTGAATTATAC	
35_CA4884	gtcagcggccgcatccctgcTAGTTGAATTAATACGGTGATTCTG	
33_CA4884	GCAATATTACAATTCCCTTCATG	
M5	ccgctgctaggcgcgccgtgACCAGTGTGATGGATATCTGC	(Noble & Johnson, 2005)
M3	gcagggatgcggccgctgacAGCTCGGATCCACTAGTAACG	(Noble & Johnson, 2005)
**Amplification of the *SAT1* marker of pSFS2A**		
3P_SAT_M5	ccgctgctaggcgcgccgtgAAATGTCGAGCGTCAAAACTAGAG	
3P_SAT_M3	gcagggatgcggccgctgacCTAGTGGATCTGAAGTTCCTATTCTC	
**Gene deletion constructs based on the *SAT1*-flipping strategy** (Reuss *et al.*, 2004)		
CaHOS2_55	gctagggccctagccgatcgCAGAATCTTGACCTGTGATTCC	
CaHOS2_53	gtcgctcgagcgacCATTTATATTAACTACTTTTCTCCTATGG	
CaHOS2_35	gctaccgcggtagcTAGTTTGTCTTGATACACATATACATATATATATA	
CaHOS2_33	gtcggagctccgaccgatcgGAAAAATGGATGCCAAGTTG	
CaSET3_55	gctagggccctagccgatcgATTGTACGAAGAAGCGGAGC	
CaSET3_53	gtcgctcgagcgacCATTAAAATAAACACTTATAAAGACTACTATC	
CaSET3_35	gctaccgcggtagcTAGTTTTTTGTTTAGAGTTTGTATATTG	
CaSET3_33	gtcggagctccgaccgatcgCAAAAGGACAATCAATTGGATG	
CaEFG1_55	gctagggccctagccgatcgAGAGACAAGCAAACAAACGACC	
CaEFG1_53	gtcgctcgagcgacCATTAATATGGGTTATATTCTTGGTAGTC	
CaEFG1_35	gctaccgcggtagcTGACTCAAGGTTCAGTTCACCC	
CaEFG1_33	gtcggagctccgaccgatcgCACCTACACGCCACAAACTATC	
CaADE2_55	gctagggccctagccgatcgCATAATTTCCTAAGCTAGTCGTGATGG	
CaADE2_53	gtcgctcgagcgacCATTATGTGTAGTGCTTGTATATGCGTG	
CaADE2_35	gctaccgcggtagcGATATCAATAGCATATTAAACAAGTAAACG	

CaADE2_33	gtcggagctccgaccgatcgAGAATCAATGCTATTGAGGGGTTAG	
**Primers used for gene complementation constructs**		
53_CA4664_comp	cacggcgcgcctagcagcggAAATACTTACAATTCTCTTCGCATC	
53_CA2580_comp	cacggcgcgcctagcagcggTTAAATTTTTAATTTATCTATCAATTGTTC	
53_CA5377_comp	cacggcgcgcctagcagcggCTAAGTCATTAGTTCTCCTAGTTTGG	
I3_SET3	gtcgctcgagcgacCTATTTCATTTTTTTATAATCAGCAAAAG	
**Quantitative Real-time PCR**		
RT5_WH11	CAGAACAATTCAAGGATAAGGTTACTG	(Zordan et al., 2006)
RT3_WH11	TTGGAGTCACCAAAAATAGCATCAG	(Zordan et al., 2006)
RT5_EFG1	CATCACAACCAGGTTCTACAACCAAT	(Zordan et al., 2006)
RT3_EFG1	CTACTATTAGCAGCACCACCC	(Zordan et al., 2006)
RT5_SAP1	TTCAACAAGATGTTGCTCAAG	(Zordan et al., 2006)
RT3_SAP1	GTTGACCGTTAGCGTAGCTC	(Zordan et al., 2006)
RT5_OP4	CCTCAAAAGCTGCTACCTC	(Zordan et al., 2006)
RT3_OP4	GTATCAACAGTTGGAGTAGAAGTAG	(Zordan et al., 2006)
RT5_PAT1	TTATCGGAATGGTCCTCGTG	(Zordan et al., 2006)
RT3_PAT1	CCAGAAGAACCATCATCAAC	(Zordan et al., 2006)
RT5_CA6009	TGGTCTTGGAAGAGCAAGAACAG	
RT3_CA6009	CTCAACACATCGGATTCAGAACC	
RT5_CA2606	CATTCCAACTTCCGATCCCAG	
RT3_CA2606	CCAATCCACCATTGATTCATCC	
RT5_CA6801	TCCAATGATGGTAGTTGGTGGAG	
RT3_CA6801	TCGGAGCCTCCCTTTGTGTC	
NRT5_CA7221	TACCTTGGGAATGGGATGTGG	
NRT3_CA7221	CCGTTGATTCAACCTTTCCTCC	
RT5_CA5377	TTCCCAGGTACAGGGTCAGTTG	
RT3_CA5377	GATTGACATCATTCAATACGCTCG	
RT5_CA4761	GATGACGATGACGAGGAAGAGG	
RT3_CA4761	GATGAGGTAAATCCCAAGATTCACC	
RT5_CA4444	TGCGACGGTGTCTTATGGTTG	
RT3_CA4444	ATCAGACCCACCAACAGTGGTG	
RT5_CA4664	TCGAAGCATCTCACAATATGGATG	
RT3_CA4664	CAGTTGTCCTAATTTCTGGCCTTG	
RT5_CA4884	GCCAACAACAACAGTAGCTCACG	
RT3_CA4884	TCCATGTGAATTACCACCACCTG	

RT5_WOR2	CAAGCACCAATTCATCTTGCC	
RT3_WOR2	TCTTTCTGCAAGTAAGACAGCCAG	

* Lower case and upper case letters denote exogenous and endogenous sequences, respectively.

Supplementary Table 4. Plasmids used in this study

Name	Parent	Target locus	Fragment*	Reference
pSFS2A	-	-		(Reuss et al., 2004)
pDH102	pSFS2A	*HOS2*	55-53 + 35-33	This study
pDH104	pSFS2A	*SET3*	55-53 + 35-33	This study
pDH106	pSFS2A	*EFG1*	55-53 + 35-33	This study
pDH108	pSFS2A	*ADE2*	55-53 + 35-33	This study
pDH112	pSFS2A	*SET3*	55-I3 + 35-33	This study

*The cloned fragments correspond to the sequences amplified by the respective primers of the listed index numbers (See Supplementary Table 3).

Supplementary Table 5. Mating frequencies

	Mating frequency	
**Strain**	**White**	**Opaque**
wt	$< 1\cdot10^{-7}$	$4.23\cdot10^{-3}$
efg1Δ/Δ	NA	$4.39\cdot10^{-3}$
efg1Δ/Δ hst2Δ/Δ	NA	$4.72\cdot10^{-3}$
efg1Δ/Δ nat4Δ/Δ	NA	$3.57\cdot10^{-3}$
efg1Δ/Δ set3Δ/Δ	$< 1\cdot10^{-7}$	$1.21\cdot10^{-3}$
efg1Δ/Δ hos2Δ/Δ	$< 1\cdot10^{-7}$	$3.79\cdot10^{-3}$
efg1Δ/Δ hst2Δ/Δ hos2Δ/Δ	$< 1\cdot10^{-7}$	$9.09\cdot10^{-4}$
efg1Δ/Δ nat4Δ/Δ set3Δ/Δ	$< 1\cdot10^{-7}$	$1.50\cdot10^{-3}$

Supplementary Table 6. Mating frequencies

	Mating frequency	
**Strain**	**White**	**Opaque**
wt	$< 1\cdot10^{-7}$	$4.23\cdot10^{-3}$
hda1Δ/Δ set3Δ/Δ	$< 1\cdot10^{-7}$	$5.67\cdot10^{-3}$
rpd31Δ/Δ set3Δ/Δ	$< 1\cdot10^{-7}$	$5.68\cdot10^{-3}$
rpd31Δ/Δ hos2Δ/Δ	$< 1\cdot10^{-7}$	$2.52\cdot10^{-3}$

Supplementary Table 7. Mating frequencies

	Mating frequency	
**Strain**	**White**	**Opaque**
wt	$< 1\cdot10^{-7}$	$4.23\cdot10^{-3}$
set1Δ/Δ hos2Δ/Δ	$< 1\cdot10^{-7}$	$1.13\cdot10^{-3}$
set1Δ/Δ set3Δ/Δ	$< 1\cdot10^{-7}$	$1.92\cdot10^{-3}$
efg1Δ/Δ set1Δ/Δ	NA	$2.41\cdot10^{-3}$
efg1Δ/Δ set1Δ/Δ hos2Δ/Δ	NA	$3.44\cdot10^{-4}$

Legends for Supplementary Tables 5, 6, 7

Quantitative mating assays were performed with an opaque phase *MTLα/α* tester strain. At least two independent experiments per genotype were performed giving qualitatively similar results. Values of one representative experiment are shown.

Supplementary Table 8. Switching frequencies

	White → Opaque		Opaque → White	
**Strain**	**Switch (%)**	**(n)**	**Switch (%)**	**(n)**
wt	3.2 ± 0.2	248	11.4 ± 1.6	178
*set3*Δ/Δ	< 0.4	266	29.2 ± 1.4	369
*set3*Δ/*SET3*	2.5 ± 0.5	236	14.2 ± 0.7	466
*hos2*Δ/*HOS2*	2.3 ± 0.1	258	3.2 ± 0.4	217
*hst2*Δ/*HST2*	1.6 ± 1.1	245	NA	-
*nat4*Δ/*NAT4*	2.4 ± 0.1	251	15.9 ± 0.8	324
*efg1*Δ/Δ *set3*Δ/*SET3*	85.8 ± 2.6	220	NA	-
*efg1*Δ/Δ *hos2*Δ/*HOS2*	87.9 ± 0.7	239	NA	-

Legends for Supplementary Table 8

Since we found a negative effect on cell viability when mutants containing the *SAT1* marker under the control of the pACT1 promoter were incubated on modified Lee´s as well as SD agar plates containing 5 µg/ml Phloxin B, the quantitative switching assays using the gene complementation mutants were performed as follows: Pure white strains were streaked out on YPD plates and incubated at room temperature for three days. Single colonies were resuspended in sterile water and spread onto YPD plates containing 5 µg/ml Phloxin B. Formation of opaque colonies or sectors was scored after 7 days. The opaque to white switching assays were performed using pure opaque colonies obtained in the white to opaque switching assays. The frequency of white colonies or colonies containing at least one white sector was scored after 5 days. For each strain, two independent experiments were carried out. The *set3*Δ/Δ mutant was included as a control. Data is shown as mean ± standard deviation.

Supplementary References

Allis, C. D., S. L. Berger, J. Cote, S. Dent, T. Jenuwien, T. Kouzarides, L. Pillus, D. Reinberg, Y. Shi, R. Shiekhattar, A. Shilatifard, J. Workman & Y. Zhang, (2007) New nomenclature for chromatin-modifying enzymes. *Cell* **131**: 633-636.

Altschul, S. F., W. Gish, W. Miller, E. W. Myers & D. J. Lipman, (1990) Basic local alignment search tool. *J Mol Biol* **215**: 403-410.

Carmen, A. A., S. E. Rundlett & M. Grunstein, (1996) *HDA1* and *HDA3* are components of a yeast histone deacetylase (HDA) complex. *J Biol Chem* **271**: 15837-15844.

Gillum, A. M., E. Y. Tsay & D. R. Kirsch, (1984) Isolation of the *Candida albicans* gene for orotidine-5'-phosphate decarboxylase by complementation of *S. cerevisiae ura3* and *E. coli pyrF* mutations. *Mol Gen Genet* **198**: 179-182.

Klar, A. J., T. Srikantha & D. R. Soll, (2001) A histone deacetylation inhibitor and mutant promote colony-type switching of the human pathogen *Candida albicans*. *Genetics* **158**: 919-924.

Landry, J., A. Sutton, S. T. Tafrov, R. C. Heller, J. Stebbins, L. Pillus & R. Sternglanz, (2000) The silencing protein Sir2 and its homologs are NAD-dependent protein deacetylases. *Proc Natl Acad Sci U S A* **97**: 5807-5811.

Lo, H. J., J. R. Kohler, B. DiDomenico, D. Loebenberg, A. Cacciapuoti & G. R. Fink, (1997) Nonfilamentous *C. albicans* mutants are avirulent. *Cell* **90**: 939-949.

Lockhart, S. R., C. Pujol, K. J. Daniels, M. G. Miller, A. D. Johnson, M. A. Pfaller & D. R. Soll, (2002) In *Candida albicans*, white-opaque switchers are homozygous for mating type. *Genetics* **162**: 737-745.

Noble, S. M. & A. D. Johnson, (2005) Strains and strategies for large-scale gene deletion studies of the diploid human fungal pathogen *Candida albicans*. *Eukaryot Cell* **4**: 298-309.

Pijnappel, W. W., D. Schaft, A. Roguev, A. Shevchenko, H. Tekotte, M. Wilm, G. Rigaut, B. Seraphin, R. Aasland & A. F. Stewart, (2001) The *S. cerevisiae* SET3 complex includes two histone deacetylases, Hos2 and Hst1, and is a meiotic-specific repressor of the sporulation gene program. *Genes Dev* **15**: 2991-3004.

Raman, S. B., M. H. Nguyen, Z. Zhang, S. Cheng, H. Y. Jia, N. Weisner, K. Iczkowski & C. J. Clancy, (2006) *Candida albicans SET1* encodes a histone 3 lysine 4 methyltransferase that contributes to the pathogenesis of invasive candidiasis. *Mol Microbiol* **60**: 697-709.

Reuss, O., A. Vik, R. Kolter & J. Morschhauser, (2004) The SAT1 flipper, an optimized tool for gene disruption in *Candida albicans*. *Gene* **341**: 119-127.

Roguev, A., D. Schaft, A. Shevchenko, W. W. Pijnappel, M. Wilm, R. Aasland & A. F. Stewart, (2001) The *Saccharomyces cerevisiae* Set1 complex includes an Ash2 homologue and methylates histone 3 lysine 4. *Embo J* **20**: 7137-7148.

Rundlett, S. E., A. A. Carmen, R. Kobayashi, S. Bavykin, B. M. Turner & M. Grunstein, (1996) *HDA1* and *RPD3* are members of distinct yeast histone deacetylase complexes that regulate silencing and transcription. *Proc Natl Acad Sci U S A* **93**: 14503-14508.

Song, O. K., X. Wang, J. H. Waterborg & R. Sternglanz, (2003) An Nalpha-acetyltransferase responsible for acetylation of the N-terminal residues of histones H4 and H2A. *J Biol Chem* **278**: 38109-38112.

Srikantha, T., L. Tsai, K. Daniels, A. J. Klar & D. R. Soll, (2001) The histone deacetylase genes *HDA1* and *RPD3* play distinct roles in regulation of high-frequency phenotypic switching in *Candida albicans*. *J Bacteriol* **183**: 4614-4625.

Tuleva, B., E. Vasileva-Tonkova & D. Galabova, (1998) A specific alkaline phosphatase from *Saccharomyces cerevisiae* with protein phosphatase activity. *FEMS Microbiol Lett* **161**: 139-144.

Xie, J., M. Pierce, V. Gailus-Durner, M. Wagner, E. Winter & A. K. Vershon, (1999) Sum1 and Hst1 repress middle sporulation-specific gene expression during mitosis in *Saccharomyces cerevisiae*. *Embo J* **18**: 6448-6454.

Zordan, R. E., D. J. Galgoczy & A. D. Johnson, (2006) Epigenetic properties of white-opaque switching in *Candida albicans* are based on a self-sustaining transcriptional feedback loop. *Proc Natl Acad Sci U S A* **103**: 12807-12812.
